# Severe Insulin Resistance Improves Immediately After Sleeve Gastrectomy

**DOI:** 10.1177/2324709615625309

**Published:** 2016-01-05

**Authors:** Rahul Sharma, Chandra Hassan, Joumana T. Chaiban

**Affiliations:** 1 Case Western Reserve University, Cleveland, OH, USA; 2 St Vincent Charity Medical Center, Cleveland, OH, USA

**Keywords:** insulin resistance, sleeve gastrectomy, HOMA-IR, type 2 diabetes

## Abstract

*Introduction*. Obese individuals exhibit insulin resistance often leading to adverse health outcomes. When compared with intensive medical therapy, bariatric surgery has shown better outcomes mainly in terms of insulin resistance and glycemic control. Using the Homeostasis Model Assessment of insulin resistance (HOMA-IR), we report herein a case illustrating a drastic improvement in severe insulin resistance after sleeve gastrectomy in the immediate postoperative period. *Case Report*. A patient with long-standing history of morbid obesity, type 2 diabetes, obstructive sleep apnea, hypertension, and severe insulin resistance (requiring approximately 2 units of insulin per kg per day) was enrolled in the medical weight management program for 6 months during which he lost 40 lbs and his insulin requirements decreased. He then underwent a sleeve gastrectomy and did not require insulin therapy as of postoperative day 1. His HOMA-IR improved by about 76% between day 1 and day 14 postoperatively. *Conclusion*. Sleeve gastrectomy leads to a drastic improvement in severe insulin resistance as early as the first postoperative day.

## Introduction

Obesity and sedentary lifestyle are major contributors to insulin resistance and type 2 diabetes (T2DM), and weight loss results in their improvement.^1^ Bariatric surgery, the most effective available tool for substantial and sustained weight loss,^2^ results in rapid amelioration of insulin resistance independent of such a weight loss early in the postoperative period. We present herein the case of a morbidly obese patient who underwent sleeve gastrectomy with subsequent immediate improvement of his severe insulin resistance. We use the Homeostasis Model Assessment of insulin resistance (HOMA-IR) to follow the progression of his insulin resistance.

## Case Report

A 49-year-old gentleman with long-standing history of morbid obesity (height 73 inches, weight 447 lbs, body mass index 59 kg/m2), T2DM, obstructive sleep apnea, and hypertension presented for weight loss management. He has had diabetes for more than 5 years, requiring insulin for at least 3 years, severely insulin resistant, on metformin 1000 mg twice a day, and a total of 415 units of insulin per day in the form of U-500 (approximately 2 units/kg/day) with a HbA1c of 7.4%. He enrolled in the medical weight management program (dietitian supervised calorie count and regular exercise) for 6 months during which he lost 40 lbs (8.9% of his initial body weight), and his insulin requirements decreased to a total of 55 units of insulin per day in the form of U-500. After that, he underwent a sleeve gastrectomy. We followed his fasting blood glucose, fasting insulin, and C-peptide levels postoperatively as illustrated in Table 1. His calculated HOMA-IR improved drastically from 18.82 on postoperative day 1 to 5.84 on postoperative day 3. Patient was kept NPO (nil per os) on the first postoperative day except for ice chips. He was then started on a full liquid diet for 2 weeks, and as outpatient transitioned to pureed diet for 2 weeks, soft diet for 2 weeks, and by 6 weeks he was on a regular diet. In the postoperative period, he required only 2 units of regular insulin subcutaneously at 1 hour after surgery (more than 15 hours away from the first fasting insulin level) and his subsequent glucose levels remained within a range of 97 to 168 mg/dL on a Q 6 hours glucose checks regimen. No further insulin was needed, and he did not require any diabetes medications on discharge. On subsequent follow-up 2 weeks later, his HOMA-IR was 4.6, and then at 7 months it was 2.4 (Table 1). He was still off of his diabetes medications. No preoperative HOMA was performed since the patient was on insulin then.

**Table 1.** Changes in Fasting Glucose, Insulin, C-Peptide, and HOMA-IR After Surgery.

	Fasting Glucose Level (mg/dL)	Fasting Insulin Level (µIU/mL)	Fasting C-Peptide (ng/mL)	HOMA-IR
Day 1^a^	154	49.5	4.9	18.82
Day 2	139	33.3	4.2	11.43
Day 3	101	23.4	3.4	5.84
Day 14	113	16.5	—	4.6
Seven months after surgery^b^	101	9.6	3	2.4

Abbreviation: HOMA-IR, Homeostasis Model Assessment of insulin resistance.

a Day 1 is defined as the next day of surgery.

b Patient not requiring any diabetes medication.

## Discussion

The HOMA-IR has been well studied and validated as a measure of insulin resistance.^3^ The widely adopted cutoff for insulin resistance is 2.6 with normal values being 1.7 to 2.5.^4^ Isbell et al^5^ showed a significant reduction in insulin resistance within 1 week after Roux en Y Gastric Bypass surgery with improvement of HOMA-IR from a baseline average of 5 ± 3.1 down to 3.3 ± 2. Rizzello et al^6^ described such an improvement as of the third postoperative day. Rao et al^7^ found an average improvement of 33.48% in insulin resistance as early as 1 to 2 weeks after surgery with a preoperative HOMA-IR range of 4.12 to 11.33. To our knowledge, this is the first case demonstrating an earlier drastic improvement in HOMA-IR after sleeve gastrectomy compared with the published literature. Our patient was also much more insulin resistant and had approximately 76% improvement in HOMA-IR from day 1 to day 14 postoperatively. No preoperative HOMA-IR was calculated since patient was on exogenous insulin, but one may assume that his HOMA-IR was even higher both at the beginning of his enrollment in the medical weight loss management and in the immediate preoperative period in view of his high insulin requirements then. Mechanisms for such an improvement have been proposed including the role of gastrointestinal hormones (GIP [gastric inhibitory peptide], GLP-1 [glucagon-like peptide-1], and Ghrelin) and various inflammatory mediators. Ghrelin plays an important physiological role in modulating insulin secretion and glucose metabolism. It has an orexigenic effect through its action on the hypothalamic appetite-regulating pathways, while in the periphery it suppresses the insulin-sensitizing hormone adiponectin, blocks hepatic insulin signaling, and inhibits insulin secretion.^8^ Karamanakos et al^9^ showed markedly reduced ghrelin levels after sleeve gastrectomy. Unfortunately, we did not measure ghrelin nor other gastric peptides. Perioperative calorie restriction, weight loss, and exercise with subsequent improvement in glucotoxicity as illustrated in the case described are also a major contributor to insulin sensitivity after bariatric surgery. This case is yet another proof of the early beneficial effect of sleeve gastrectomy on insulin resistance, an effect that is more pronounced if paired with perioperative lifestyle interventions and weight loss.
